# A clinical pharmacokinetic drug-drug interaction study between dextromethorphan and emvododstat, a potent anti-SARS-CoV-2 dihydroorotate dehydrogenase inhibitor

**DOI:** 10.1007/s00228-023-03513-4

**Published:** 2023-06-06

**Authors:** Terri L. Morton, Oscar L. Laskin, Diksha Kaushik, Lucy Lee, Jiyuan Ma, Cristian M. Bar, Allan Kristensen, Kylie O’Keefe, Lee Golden, Matthew Klein, Ronald Kong

**Affiliations:** 1 PTC Therapeutics, 100 Corporate Court, South Plainfield, NJ 07080 USA; 2 Syneos Health, Quebec, QC Canada

**Keywords:** Emvododstat, SARS-CoV-2, COVID-19, DHODH, Pharmacokinetics, CYP2D6 Inhibitor

## Abstract

**Purpose:**

A therapeutic agent that targets both viral replication and the hyper-reactive immune response would offer a highly desirable treatment for severe acute respiratory syndrome corona virus 2 (SARS-CoV-2, coronavirus disease 2019, COVID-19) management. Emvododstat (PTC299; 4-chlorophenyl 6-chloro-1-[4-methoxyphenyl]-1,3, 4,9-tetrahydro-2H-pyrido[3,4-b]indole-2-carboxylate) was found to be a potent inhibitor of immunomodulatory and inflammation-related processes by inhibition of dihydroorotate dehydrogenase to reduce the severity of SARS-CoV-2 infections This drug interaction study was performed to determine if emvododstat was an inhibitor of CYP2D6.

**Methods:**

Potential drug-drug interactions between emvododstat and a CYP2D6 probe substrate (dextromethorphan) were investigated by measuring plasma dextromethorphan and metabolite (dextrorphan) concentrations before and after emvododstat administration. On day 1, 18 healthy subjects received an oral dose of 30 mg dextromethorphan followed by a 4-day washout period. On day 5, subjects received an oral dose of 250 mg emvododstat with food. Two hours later, 30 mg dextromethorphan was administered.

**Results:**

When given with emvododstat, plasma dextromethorphan concentrations increased substantially, while metabolite levels (dextrorphan) remained essentially the same. Maximum plasma dextromethorphan concentration (*C*_max_) increased from 2006 to 5847 pg/mL. Dextromethorphan exposure (AUC) increased from 18,829 to 157,400 h·pg/mL for AUC_0-last_ and from 21,585 to 362,107 h·pg/mL for AUC_0-inf_ following administration of emvododstat. When dextromethorphan parameters were compared before and after emvododstat, least squares mean ratios (90% confidence interval) were found to be 2.9 (2.2, 3.8), 8.4 (6.1, 11.5), and 14.9 (10.0, 22.1) for C_max_, AUC_0-last_, and AUC_0-inf_, respectively.

**Conclusion:**

Emvododstat appears to be a strong CYP2D6 inhibitor. No drug-related treatment emergent adverse effects (TEAEs) were considered to be severe or serious.

**Trial registration:**

EudraCT 2021-004626-29, 11 May 2021.

## Introduction

Emvododstat (PTC299; 4-chlorophenyl 6-chloro-1-[4-methoxyphenyl]-1,3, 4,9-tetrahydro-2H-pyrido[3,4-b]indole-2-carboxylate) is an orally available inhibitor of the de novo pyrimidine synthesis enzyme dihydroorotate dehydrogenase (DHODH) [1, 2]. Inhibition of DHODH results in the depletion of pyrimidine nucleotides in rapidly dividing cells, leading to arrest of cell growth and DNA replication (G1/S-phase cell cycle arrest) and subsequent differentiation or cell death [3]. Cells may utilize salvage pathways whereby pyrimidine nucleotides are synthesized from intermediates formed from the degradation of RNA and DNA taken into the cell [2, 4, 5]. Normal cells typically do not rely on de novo pyrimidine biosynthesis and utilize the salvage pathway to provide pyrimidine nucleotides. Rapidly dividing cells rely on a de novo pathway [2]. Consistent with this, emvododstat does not impair the viability or proliferation of cultured human bone marrow progenitor cells and has shown no evidence of myelotoxicity in toxicity studies in rats and dogs dosed for up to 28 days or in humans [2].

Inhibition of de novo pyrimidine biosynthesis has been identified as a mechanism to inhibit viral replication, including replication of the RNA virus severe acute respiratory syndrome corona virus 2 (SARS-CoV-2) [6, 7]. Further, lethality associated with SARS-CoV-2 infection has been associated with excessive activation of the immune response [7]. Emvododstat has a dual mechanism of action, which inhibits SARS-CoV-2 replication and attenuates the production of pro-inflammatory cytokines [2]. In vitro, cell culture assays indicate a decreased capsid protein (EC_50_ 1.96 nM) and decreased viral titer (EC_50_ 2.6 nM, EC_90_ 53 nM) of SARS-CoV-2019 following cell exposure to emvododstat [2]. This dual mechanism of action together with a well-established safety profile as demonstrated in previous completed clinical studies [8] supports an appropriate benefit/risk profile for evaluating the drug in subjects with coronavirus disease 2019 (COVID-19) with a dose regimen of 200 mg BID for 7 days followed by 50 mg QD for another 7 days.

In vitro CYP inhibition potential was assessed in human liver microsomes for emvododstat and its primary *O*-desmethyl metabolite up to the maximum solubility for both. Emvododstat was identified as a non-competitive inhibitor of CYP2D6 with an inhibition constant (*K*_*i*_) of 37.7 nM, indicating a need for clinical confirmation [9]. The purpose of the current study was to investigate potential drug-drug interactions (DDIs) between emvododstat and other medications that are metabolized by CYP2D6. Dextromethorphan is a CYP2D6 substrate and is recognized as a CYP2D6 marker substrate for the assessment of CYP2D6 inhibition potential [10]. It is an antitussive drug and is used for temporary relief of coughs that are caused by certain infections of the air passages. In addition, dextromethorphan may be co-administered with emvododstat in clinical studies in COVID-19 subjects and potentially in clinical practice in the future. Dextromethorphan is given every 4 to 12 h as needed. As such, this study was designed to evaluate the maximum effect of emvododstat on the pharmacokinetics (PK) of dextromethorphan if there is any.

### Methods

#### Participants

The study was conducted in accordance with the principles outlined in the 1964 Declaration of Helsinki and its later amendments and the International Conference on Harmonisation Good Clinical Practice. The Independent Ethics Committee (Evaluation of Ethics in Biomedical Research) reviewed and approved the clinical study protocol, any clinical study protocol amendments, subject information sheets, written informed consent forms, and other relevant documentation. Before participating in the study, each subject was apprised of the nature and purpose of the study, and written informed consent was obtained.

Subjects were healthy, non-smokers with body mass index (BMI) between 18 and 30 kg/m^2^ and weight ranging from 50 to 110 kg. Subjects were instructed to use only approved contraceptives; other prescribed medicines were stopped 30 days prior to the first dose. Methylxanthine-containing beverages, grapefruit juice, and herbal supplements were stopped 48 h, 72 h, and 14 days prior to dosing, respectively. All subjects were genotyped for CYP2D6 status, and poor metabolizers (PMs) were excluded. Subjects were in good health, signed an informed consent form, and did not participate in other trials within 30 days of dosing.

#### Formulation

PTC299 (4-chlorophenyl 6-chloro-1-[4-methoxyphenyl]-1,3, 4,9-tetrahydro-2H-pyrido[3,4-b]indole-2-carboxylate) is a proprietary tablet provided by PTC Therapeutics (South Plainfield, NJ, USA). Commercially available dextromethorphan hydrobromide monohydrate 40.2 mg equivalent to 30 mg dextromethorphan (DARO Retard capsules) was purchased by PRA Health Sciences (ICON, Netherlands).

### Study design and conduct

On day 1, 18 healthy subjects (ages ≥ 18 and ≤ 65 years) received a single oral dose of 30 mg (capsule) dextromethorphan followed by a 4-day washout period. On day 5, subjects received a single oral dose of 250 mg emvododstat with food. Two hours later, subjects were administered a single oral dose of 30 mg modified-release dextromethorphan.

Blood samples (3 mL) for dextromethorphan and dextrorphan assessments were collected in K_2_EDTA vacutainers on day 1 (dextromethorphan alone) and on day 5 (dextromethorphan in combination with emvododstat) at the following timepoints: 0- (predose), 0.5-, 1-, 2-, 3-, 4-, 5-, 6-, 8-, 12-, 24-, and 48-h post dextromethorphan dose. Within 120 min of collection of blood samples, plasma was harvested by centrifuging blood samples at 2000 ± 20 g for 10 min at 4 °C. Upon harvesting, plasma was transferred to 2-mL polypropylene tubes and immediately stored at – 20 ± 5 °C until analysis.

The safety and tolerability of dextromethorphan alone and with emvododstat were monitored.

#### Bioanalytical method

Concentrations of dextromethorphan and dextrorphan in human plasma samples were determined using high-performance liquid chromatography with tandem mass spectrophotometric (LC-MS/MS) detection. The bioanalytical method was validated, and its performance during bioanalysis was within the limits specified in current regulatory guidance over a calibration range of 50 to 50,000 pg/mL. Validation included testing such as linearity, sensitivity, specificity, selectivity, accuracy and precision, dilution effects, recovery, ruggedness, and stability.

Briefly, dextromethorphan and dextrorphan were extracted from 0.2 mL of human K_2_EDTA plasma using an automated liquid–liquid extraction followed by detection using LC-MS/MS. Stable isotope labeled internal standard (dextromethorphan-d3 and dextrorphan-d3) was used to track the analytes potential variability. To 0.2 mL of plasma, the internal standard (IS) working solution diluted in an ammonium formate/ammonium hydroxide buffer solution was added. The analytes and ISs were extracted using methyl-tert-butyl-ether (MTBE). A portion of the upper layer was transferred to a 96 deep well collection plate using an automated liquid handling system. After evaporation to dryness, the dry residue was reconstituted with the mobile phase and injected on a Sciex API 4000. The chromatographic separation was performed in isocratic mode using BetaSil CN column (dimensions 50 × 4.6 mm, 5 μm) and methanol/Milli-Q type water with ammonium formate and formic acid as mobile phase. The mass spectrometer detection was performed in positive ion mode with multiple reaction monitoring (MRM) transitions of 272.2 → 215.3, 275.2 → 215.3, 258.2 → 201.2, and 261.2 → 157.2 for dextromethorphan, dextromethorphan-d3, dextrorphan, and dextrorphan-d3, respectively.

Dextromethorphan and dextrorphan concentrations in study plasma samples were determined using duplicate plasma calibration curves of dextromethorphan and dextrorphan in each analytical batch. Additionally, duplicate quality control plasma samples spiked with dextromethorphan and dextrorphan at four concentration levels (low, 150 pg/mL; intermediate, 2000 pg/mL; medium, 25,000 pg/mL; and high, 375,000 pg/mL) were included in each analytical batch to assess the assay performance of the bioanalytical method. The accuracy (%bias) of QC samples in all analytical batches was within ± 4.6%, and precision (%coefficient of variation) was less than 6.2% for dextromethorphan and dextrorphan. The method performance was further demonstrated with 100% passing rate during the conduct of incurred sample reproducibility (ISR) assessment.

### Pharmacokinetic analysis

Non-compartmental pharmacokinetics were performed using Phoenix^®^ WinNonlin (Version 8.1, Certara, Princeton, NJ, USA). Primary parameters consisted of AUC_0-last_, AUC_0-inf_, and *C*_max_. Secondary parameters included *T*_1/2_, *T*_max_, and CL/F (drug clearance). Bioavailability assessments were calculated using SAS software (Version 9.4, SAS, Cary, NC, USA) in accordance with current regulatory guidelines [11, 12]. A mixed linear model was fitted on log-transformed AUC_0-inf_, AUC_0-t_, and C_max_ for both parent and metabolite. The model included terms for treatment (combined emvododstat + dextromethorphan as test treatment versus dextromethorphan as reference treatment) and subject as a random factor. LSM (geometric least squares mean) for the treatment ratio and 90% confidence intervals (90% CI) were estimated from the model.

## Results

### Subject characteristics and safety

A total of 5 male subjects and 13 female subjects aged between 18 and 42 years and with a BMI between 19.3 and 28.5 kg/m^2^ participated in the study. Subjects having the PM CYP2D6 genotype were excluded from the study. The numbers of dosed subjects with each CYP2D6 genotype were 1 ultra-rapid metabolizer, 6 intermediate metabolizers, and 11 normal metabolizers.

All randomized subjects (18) completed the study. Single doses of dextromethorphan with and without emvododstat resulted in drug-related AEs (adverse effects) that were mild and reversible. All treatment emergent adverse effects (TEAEs) resolved, and there were no serious adverse effects.

### Pharmacokinetics

**Fig. 1:**
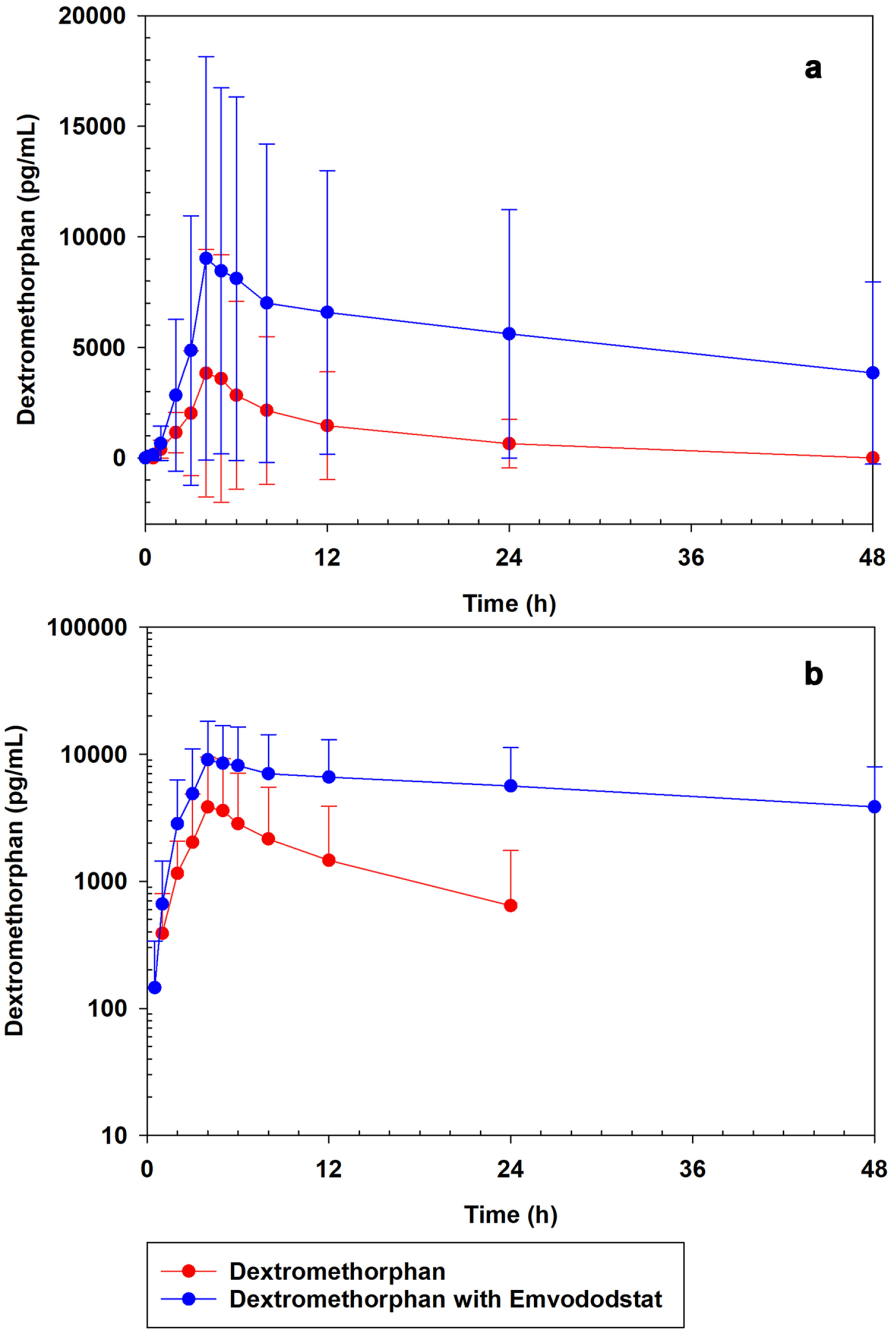
Mean (SD) plasma dextromethorphan concentration when administered separately (30 mg) and in combination with emvododstat (250 mg). **a** Linear. **b** Log-linear. Red circles, dextromethorphan; blue circles, dextromethorphan with emvododstat

**Fig. 2:**
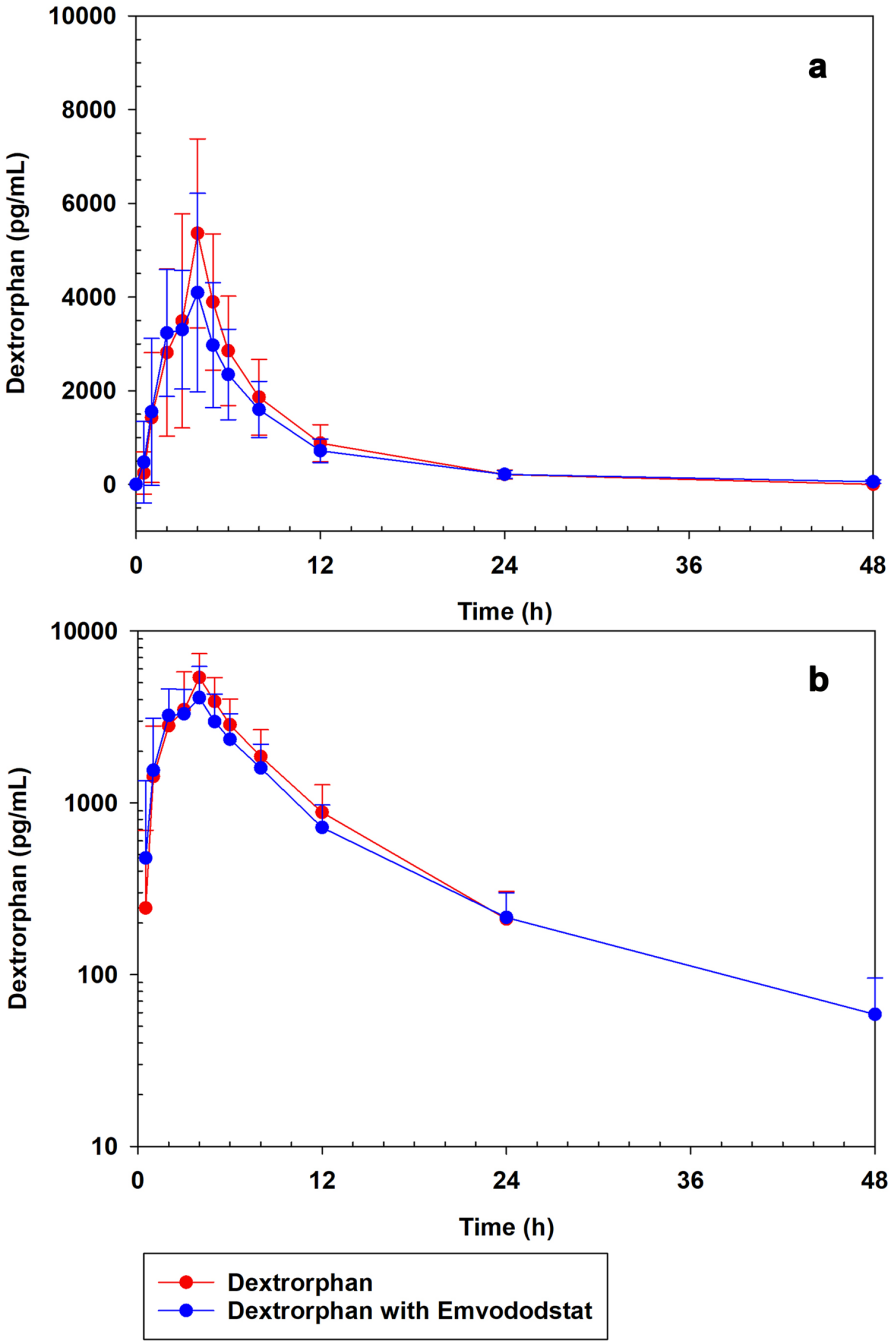
Mean (SD) plasma dextrorphan concentration when dextromethorphan (30 mg) is administered separately and in combination with emvododstat (250 mg). **a** Linear. **b** Log-linear. Red circles, dextromethorphan; blue circles, dextromethorphan with emvododstat

Dextromethorphan and dextrorphan plasma concentrations were measured at timepoints after dosing on Day 1 and on Day 5 after coadministration with emvododstat. Both dextromethorphan and dextrorphan plasma concentrations increased rapidly with maximum concentrations (*C*_max_) observed at 4-h post dose in the presence and absence of emvododstat (Figs. 1 and 2). The mean half-life (*T*_1/2_) of plasma dextromethorphan concentrations on day 1 was estimated at 9 h. In the presence of emvododstat on day 5, dextromethorphan showed a prolonged *T*_1/2_ of 41 h. Dextrorphan (alone and in combination with emvododstat) had effective *T*_1/2_ values of 6 to 8 h.

### Dextromethorphan

Peak exposure (*C*_max_) and total systemic exposure (AUC_0-last_ and AUC_0-inf_) to dextromethorphan alone and with emvododstat were compared to evaluate potential DDIs. Dextromethorphan levels were higher after coadministration with emvododstat on day 5 compared to day 1. Maximum geometric mean dextromethorphan concentrations were 2006 pg/mL for dextromethorphan alone compared to 5847 pg/mL in the presence of emvododstat (Table 1). The geometric LSM ratio was 2.9 for *C*_max_ (Table 2).

**Table 1:** Summary of pharmacokinetic parameters for dextromethorphan with and without emvododstat (geometric mean, %CV)

Treatment	*n*	*C*_max_ (pg/mL)	*T*_max_^a^ (h)	AUC_0-last_ (h·pg/mL)	AUC_0-inf_ (h·pg/mL)	*T*_1/2_ (h)	CL/F (L/h)
Dextromethorphan	18	2006 (180.8)	4 (2–5)	18,829 (199.0)	21,585 (176.7)	9.15 (25.2)	1367 (176.7)
Dextromethorphan with emvododstat	18	5847 (141.3)	4 (4–24)	157,400 (154.6)	362,107^b^ (117.0)	41.4^b^ (39.2)	81.5^b^ (117.0)

*C*_*max*_ maximum observed concentration, *T*_*max*_ time of *C*_max_, *AUC*_*0-last*_ area under the dextromethorphan curve from 0 to the last measurable timepoint, *AUC*_*0-inf*_ area under the dextromethorphan curve from 0 extrapolated to infinity, *T*_*1/2*_ apparent elimination half-life, *CL/F* total drug clearance corrected for fraction of dose absorbed

^a^Median (range)

^b^*n* = 16

**Table 2:** Statistical analysis of pharmacokinetic parameters for dextromethorphan

Treatment comparison (test vs reference)	PK parameter	Geometric LS mean ratio	90% confidence interval
Dextromethorphan with emvododstat (*T*) Dextromethorphan (*R*)	*C*_max_	2.914	2.218, 3.828
	AUC_0-t_	8.359	6.084, 11.485
	AUC_0-inf_	14.878	10.012, 22.108

*LS Mean* least squares mean, *T* test, *R* reference

The mean total exposure to dextromethorphan (AUC) in the presence emvododstat increased significantly compared to dextromethorphan alone. Geometric mean AUC_0-last_ values went from 18,829 h·pg/mL (dextromethorphan) to 157,400 h·pg/mL (dextromethorphan + emvododstat), while AUC_0-inf_ went from 21,585 to 362,107 h·pg/mL (Table 1).

These increases resulted in geometric LSM ratios of 8 and 15 for AUC_0-last_ and AUC_0-inf_, respectively, when dextromethorphan alone was compared to dextromethorphan in the presence of emvododstat (Table 2).

### Dextrorphan

Exposure to the major metabolite of dextromethorphan, dextrorphan, was evaluated both when the parent drug was given alone and with emvododstat. Geometric mean *C*_max_ values for dextrorphan when dextromethorphan was given alone or with emvododstat were 4999 and 4054 pg/mL, respectively. Overall exposures (AUC_0-last_ and AUC_0-inf_) to dextrorphan when 30 mg dextromethorphan capsules were administered alone were 31,852 and 33,194 pg/mL, respectively. Dextromethorphan given concurrently with emvododstat resulted in dextrorphan AUC_0-last_ and AUC_0-inf_ values of 29,382 and 30,875 pg/mL, respectively (Table 3).

**Table 3:** Summary of pharmacokinetic parameters for dextrorphan with and without emvododstat (geometric mean, %CV)

Treatment	*n*	*C*_max_ (pg/mL)	*T*_max_^a^ (h)	AUC_0-last_ (h·pg/mL)	AUC_0-inf_ (h·pg/mL)	*T*_1/2_ (h)
Dextrorphan	18	4999 (50.6)	4 (2–4)	31,852 (45.7)	33,194 (43.8)	5.86 (30.1)
Dextrorphan with emvododstat	18	4054 (52.1)	4 (1–4)	29,382 (40.7)	30,875 (38.8)	8.41 (49.6)

*C*_*max*_ maximum observed concentration, *T*_*max*_ time of *C*_max_, *AUC*_*0-last*_ area under the dextromethorphan curve from 0 to the last measurable timepoint, *AUC*_*0-inf*_ area under the dextromethorphan curve from 0 extrapolated to infinity, *T*_*1/2*_ apparent elimination half-life, *CL/F* total drug clearance corrected for fraction of dose absorbed

^a^Median (range)

### Metabolic ratios

Geometric mean metabolite/parent ratios for AUC were 1.69 prior to administration of emvododstat. Following administration of dextromethorphan in the presence of emvododstat, dextrorphan/dextromethorphan geometric mean AUC ratios declined to 0.182. Overall exposure (AUC) to dextromethorphan given concomitantly with emvododstat compared to dextromethorphan alone showed a ratio of 8.36.

## Discussion

A therapeutic agent that targets both viral replication and the hyper-reactive immune response would offer a highly desirable treatment for SARS-CoV-2 (COVID-19) management. Emvododstat (PTC299) was found to be a potent inhibitor of immunomodulatory and inflammation-related processes by inhibition of DHODH to reduce SARS-CoV-2 replication in a cell-based assay [1, 2].

Dextromethorphan is used as an antitussive in over-the-counter preparations. Dextromethorphan is a sigma-1 receptor agonist and an uncompetitive NMDA (N-methyl-D-aspartate) receptor antagonist [13]. It is also commonly used as a marker for CYP2D6 in in vivo DDI studies [10]. The marketed dextromethorphan quinidine combination is indicated for the treatment of pseudobulbar affect [13].

In vitro, emvododstat and, to a lesser extent, *O*-desmethyl emvododstat were shown to inhibit CYP2D6 activity (IC_50_ 0.017 µM and K_i_ 38 nM) [9]. The current study was performed to see if emvododstat functioned as an inhibitor of CYP2D6 in vivo. A single dose of 250 mg emvododstat was chosen because this was the clinical dose used in efficacy studies.

Potential DDIs between emvododstat and a CYP2D6 substrate (dextromethorphan) were investigated by measuring concentrations of plasma dextromethorphan and its major metabolite, dextrorphan, before and after emvododstat administration. As this was a CYP2D6 inhibition study, only the victim drug and its metabolite were measured in plasma, and emvododstat concentrations were not determined.

The rate of metabolism of dextromethorphan decreased significantly, leading to a 94% reduction in clearance (CL/F) and a 32-h increase in *T*_1/2_. Consequently, maximum plasma dextromethorphan concentrations increased substantially in the presence of emvododstat. Geometric LSM ratios for dextromethorphan *C*_max_ and AUC_0-inf_ were 2.9 and 14.9, respectively.

The metabolic ratio (AUC_metabolite_/AUC_parent_) exhibited a large decrease due to increased plasma concentrations of the parent compound when given concomitantly with emvododstat (0.18). Principally, a decrease in the metabolic ratio was considered as inhibition of CYP2D6 which forms the metabolite [14]. This is further illustrated by a ratio of dextromethorphan AUC with/without emvododstat of 8.4.

It was expected that dextrorphan levels would decrease when dextromethorphan levels increased with CYP2D6 inhibition. However, there appeared to be no effect on overall exposure to dextrorphan since LSM ratios and 90% CIs were between 0.80 and 1.25 [12]. Peak exposure was slightly affected by CYP2D6 inhibition; the *C*_max_ LSM ratio was just inside, and 90% CIs fell outside of the no effect range. No other metabolites from an alternate pathway were measured, such as the major metabolite of dextrorphan, dextrorphan-O-glucuronide catalyzed by UGT2B, and the minor metabolite, 3-hydroxymorphinan, catalyzed by CYP3A4. The formation of metabolites could lead to the decreased AUC_metabolite_/AUC_parent_ ratio due to decreases in dextrorphan concentrations by metabolism [13–15].

Quinidine inhibits CYP2D6 activity, increasing bioavailability of dextromethorphan concentrations in the central nervous system compared to dextromethorphan alone [16]. Our data agree with those from a combination product dextromethorphan and quinidine. Quinidine has been shown to convert subjects to a PM phenotype with doses of dextromethorphan up to 45 mg [13]. Other researchers have found a similar result of no change in the metabolite, dextrorphan, after inhibition of the metabolism of dextromethorphan by quinidine [13, 16].

These results show that emvododstat is a strong inhibitor of CYP2D6 activity, and other drugs that are metabolized through CYP2D6 may be affected when given concomitantly and may require dose adjustment.

## Conclusions

Emvododstat is a strong inhibitor of CYP2D6 activity as demonstrated using dextromethorphan as a probe to represent a sensitive substrate drug. As such, a clinically significant interaction would be expected if emvododstat was used concurrently with compounds that relied on CYP2D6 as a major pathway of biotransformation. Dosages of drugs that show a narrow margin of safety and are CYP2D6 substrates may need to be adjusted if given with emvododstat. One should try to avoid the concomitant use of CYP2D6 substrates in subjects receiving emvododstat.

## Data Availability

All data are proprietary property of PTC Therapeutics.
